# Elevated levels of damage-associated molecular patterns HMGB1 and S100A8/A9 coupled with toll-like receptor-triggered monocyte activation are associated with inflammation in patients with myelofibrosis

**DOI:** 10.3389/fimmu.2024.1365015

**Published:** 2024-09-25

**Authors:** Geraldine De Luca, Nora P. Goette, Paola R. Lev, Maria C. Baroni Pietto, Cecilia P. Marin Oyarzún, Miguel A. Castro Ríos, Beatriz Moiraghi, Federico Sackmann, Laureano J. Kamiya, Veronica Verri, Victoria Caula, Vanina Fernandez, Angeles Vicente, Julio Pose Cabarcos, Vanesa Caruso, Maria F. Camacho, Irene B. Larripa, Marina Khoury, Rosana F. Marta, Ana C. Glembotsky, Paula G. Heller

**Affiliations:** ^1^ División Hematología Investigación, Instituto de Investigaciones Médicas Alfredo Lanari, Facultad de Medicina, Universidad de Buenos Aires (UBA), Buenos Aires, Argentina; ^2^ Instituto de Investigaciones Médicas (IDIM), UBA-Consejo Nacional de Investigaciones Científicas y Técnicas (CONICET), Buenos Aires, Argentina; ^3^ Consultorios Hematológicos, Buenos Aires, Argentina; ^4^ Hospital Ramos Mejía, Buenos Aires, Argentina; ^5^ Centro de Hematologia Pavlovsky, Fundaleu, Buenos Aires, Argentina; ^6^ División Hematología Clínica, Instituto de Investigaciones Médicas Alfredo Lanari, Facultad de Medicina, Universidad de Buenos Aires, Buenos Aires, Argentina; ^7^ Departamento de Hematología, Hospital Posadas, Buenos Aires, Argentina; ^8^ Departamento de Hematología, Hospital Alemán, Buenos Aires, Argentina; ^9^ Departamento de Hematología, Sanatorio Otamendi Miroli, Buenos Aires, Argentina; ^10^ Departamento de Hematología, Hospital Piñero, Buenos Aires, Argentina; ^11^ Laboratorio de Genética Hematológica, Instituto de Medicina Experimental, IMEX-CONICET/Academia Nacional de Medicina, Buenos Aires, Argentina; ^12^ Departamento de Docencia e Investigación, Instituto de Investigaciones Médicas Alfredo Lanari, Facultad de Medicina, Universidad de Buenos Aires, Buenos Aires, Argentina

**Keywords:** myelofibrosis, monocyte, inflammation, HMGB1, S100A8/A9, Toll-like receptors

## Abstract

Inflammation plays a pivotal role in the pathogenesis of primary and post-essential thrombocythemia or post-polycythemia vera myelofibrosis (MF) in close cooperation with the underlying molecular drivers. This inflammatory state is induced by a dynamic spectrum of inflammatory cytokines, although recent evidence points to the participation of additional soluble inflammatory mediators. Damage-associated molecular patterns (DAMPs) represent endogenous signals released upon cell death or damage which trigger a potent innate immune response. We assessed the contribution of two prototypical DAMPs, HMGB1 and S100A8/A9, to MF inflammation. Circulating HMGB1 and S100A8/A9 were elevated in MF patients in parallel to the degree of systemic inflammation and levels increased progressively during advanced disease stages. Patients with elevated DAMPs had higher frequency of adverse clinical features, such as anemia, and inferior survival, suggesting their contribution to disease progression. Monocytes, which are key players in MF inflammation, were identified as a source of S100A8/A9 but not HMGB1 release, while both DAMPs correlated with cell death parameters, such as serum LDH and cell-free DNA, indicating that passive release is an additional mechanism leading to increased DAMPs. HMGB1 and S100A8/A9 promote inflammation through binding to Toll-like receptor (TLR) 4, whereas the former also binds TLR2. Monocytes from MF patients were shown to be hyperactivated at baseline, as reflected by higher CD11b and tissue factor exposure and increased expression levels of proinflammatory cytokines IL-1β and IL-6. Patient monocytes showed preserved TLR4 and TLR2 expression and were able to mount normal or even exacerbated functional responses and cytokine upregulation following stimulation of TLR4 and TLR2. Elevated levels of endogenous TLR ligands HMGB1 and S100A8/A9 coupled to the finding of preserved or hyperreactive TLR-triggered responses indicate that DAMPs may promote monocyte activation and cytokine production in MF, fueling inflammation. Plasma IL-1β and IL-6 were elevated in MF and correlated with DAMPs levels, raising the possibility that DAMPs could contribute to cytokine generation *in vivo*. In conclusion, this study highlights that, in cooperation with classic proinflammatory cytokines, DAMPs represent additional inflammatory mediators that may participate in the generation of MF inflammatory state, potentially providing novel biomarkers of disease progression and new therapeutic targets.

## Graphical Abstract

Elevated levels of damage-associated molecular patterns (DAMPs), HMGB1 and S100A8/A9, in the circulation of patients with myelofibrosis (MF) may trigger Toll-like receptor (TLR) activation in monocytes promoting inflammatory responses, such as CD11b and tissue factor expression, and production of proinflammatory cytokines, such as IL-1β and IL-6. Levels of proinflammatory mediators, including DAMPs (HMGB1, S100A8/A9) and cytokines (IL-1β and IL-6) parallel disease progression and the degree of systemic inflammation. Created with BioRender.com.

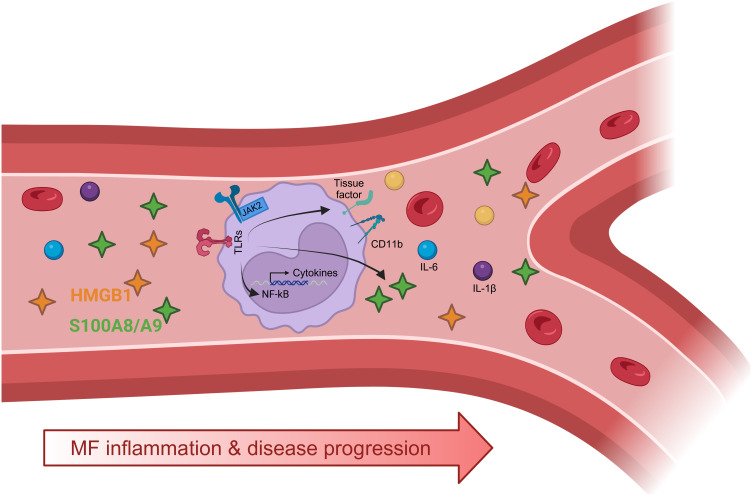

## Introduction

The role of inflammation in the pathogenesis and progression of myeloid neoplasms is being increasingly recognized and represents a hallmark of chronic myeloproliferative neoplasms (MPN) (1–4), which are stem cell disorders characterized by overproduction of myeloid progenitors and mature blood cells. Inflammation contributes to all MPN phenotypes, including essential thrombocythemia (ET) and polycythemia vera (PV), although it is most prominent in patients with primary or post-ET/PV myelofibrosis (MF), where it has been shown to be associated with worse outcome (5, 6). Myelofibrosis is driven by clonal proliferation of myeloid and megakaryocyte progenitors which promote bone marrow fibrosis through the release of profibrotic and proinflammatory growth factors (7). As disease progresses, ineffective hematopoiesis leads to anemia and thrombocytopenia and extramedullary hematopoiesis results in progressive splenomegaly, which together with constitutional symptoms has an adverse impact on patient quality of life. Despite significant advances in recent years and novel therapeutic combinations, survival remains poor in a substantial proportion of MF patients (8). Driver mutations in *JAK2*, *CALR* or *MPL* are present in most but not all MF patients, with 10-15% lacking all three mutations, referred as triple-negative patients. Sequential acquisition of cooperating mutations in high-molecular risk genes (HMR), such as *ASXL1*, *SRSF2*, *U2AF1*, *IDH1/2* and *EZH2*, promote clonal evolution and disease transformation, with increased risk of acute leukemia (9). In close cooperation with intrinsic clonal mutations, cell-extrinsic inflammatory signals contribute to the maintainance and propagation of the myeloproliferative clone, favouring clonal instability and promoting bone marrow fibrosis. This inflammatory state is driven by a growing spectrum of proinflammatory cytokines secreted mainly by monocytes, although hematopoietic progenitors, megakaryocytes and other blood cells represent additional sources of cytokine release (5, 10, 11). Parallel to dysregulated JAK2-STAT signaling, the NF-κB pathway is hyperactivated (12, 13), which provides key inflammatory cues that reinforce cytokine production, whereas both signaling pathways cooperate to elicit the activation of blood cells, including monocytes (11).

In addition to the well-established role of inflammatory cytokines, growing evidence supports the participation of additional soluble mediators in the generation and maintainance of MPN inflammatory state (14, 15). Damage-associted molecular patterns (DAMPs), also known as alarmins, are host danger signals which include a wide array of endogenous ligands released upon cell damage or death which elicit the innate imune response through stimulation of pattern recognition receptors, thus mimicking the action of pathogen-associated molecular patterns (PAMPs) derived from microorganisms (16). High mobility group box-1 protein (HMGB1), probably the first alarmin to be identified, and S100A8/A9 represent prototypes of DAMPs involved in several disease states characterized by sterile inflammation, such as autoimmune disorders, diabetes and cancer (17–19). HMGB1 is an ubiquitously expressed nuclear DNA-binding protein that contributes to chromatin stability and transcriptional regulation under physiological conditions (18). S100A8 and S100A9 members of the S100 Ca^2+^-binding protein family are highly expressed as heterodimeric S100A8/A9 complexes in the cytosol of neutrophils and monocytes, where they constitute up to 40% and 5% of neutrophil and monocyte protein content, respectively. Under steady state conditions, the heterodimeric S100A8/A9 complex regulates several neutrophil functions and exerts antimicrobial activities through its ion chelation properties (19, 20).

During inflammation, HMGB1 and S100A8/A9 are actively secreted from immune cells, including monocytes, macrophages, neutrophils and activated platelets, to the extracellular space, where they behave as alarmins, eliciting a potent immune response (21). Leakage of HMGB1 and S100A8/A9 to the extracellular compartment may also be due to passive release after different forms of cell death, including lytic cell death, netosis and pyroptosis, an inflammasome-dependent proinflammatory type of cell death (22). Extracellular HMGB1 and S100A8/A9 promote monocyte activation and tissue factor expression (23, 24) and trigger NF-kB mediated transcriptional upregulation and release of several proinflammatory cytokines from monocytes and macrophages (23, 25). Moreover, these alarmins induce NLRP3 inflammasome assembly, which drives caspase-1-mediated IL-1β and IL-18 processing and release and triggers pyroptotic cell death through the formation of Gasdermin D pores leading to leakage of inflammatory proteins, including DAMPs, to the microenvironment (22). Both DAMPs promote neutrophil, platelet and endothelial activation, while HMGB1 is a potent inducer of neutrophil extracellular trap release (23, 26), overall amplifying the inflammatory response and contributing to immunothrombosis. In addition, HMGB1 has been implicated in a variety of profibrotic conditions, such as pulmonary, liver and cardiac fibrosis (27). Among pattern recognition receptors (PRR), Toll-like receptors (TLR) are the main receptors involved in DAMP recognition. In particular, HMGB1 and S100A8/A9 signal through TLR4 whereas the former also binds TLR2 (18, 20, 21). In addition, both DAMPs may signal through receptor for advanced glycation endproducts (RAGE) (18, 20, 21).

In this work, we studied the participation of two alarmins, HMGB1 and S100A8/A9, in MF inflammatory state and the contribution of TLR-triggered inflammatory responses to monocyte activation in MF patients.

## Patients and methods

### Patients

The study included 50 patients with MF, comprising overt primary myelofibrosis, post-ET and post-PV myelofibrosis, diagnosed according to the 2016 World Health Organization (WHO) criteria (28) or the International Working Group on Myelofibrosis Research and Treatment (IWG-MRT) criteria (29), after written informed consent. The study was approved by the Instituto de Investigaciones Médicas Alfredo Lanari Ethics Committee. Clinical features of MF patients are described in **Table 1**. Age of MF patients and controls (25 healthy individuals) was 66 (19-88) and 60 (32-83) years old, respectively, while 60% in both groups were women. In addition, we included patients with ET (n=15) and PV (n=15) diagnosed according to 2016 WHO criteria (28), as detailed in **Supplementary Table 1**. Exclusion criteria included acute or chronic infection, thrombotic events, other neoplasms or autoimmune disease at the time of the study.

**Table 1:** Features of patients with myelofibrosis.

	Patients (n=50)
Age (years), median (range)	66 (19-88)
Female, n (%)	30 (60%)
Myelofibrosis, n (%)	
Primary	35 (70%)
post-ET	7 (14%)
post-PV	8 (16%)
Driver mutation, n (%)	
*JAK2*V617F	28 (56%)
*CALR*	14 (28%)
*MPL*	4 (8%)
Triple-negative	4 (8%)
High molecular risk mutation,* n (%)	
*ASXL1*	16 (32%)
*SRSF2*	4 (8%)
*IDH1/2*	1 (2%)
*U2AF1*	1 (2%)
Unfavourable karyotype** (n evaluable=28), n (%)	3 (11%)
Hemoglobin (gr/dL), median (range)	9.9 (6.3-17.8)
Hemoglobin <10 gr/dL, n (%)	25 (50%)
Transfusion dependency, n (%)	10 (20%)
Platelet count (x 10^9^/L), median (range)	276.5 (21-831)
Platelet count <100 x 10^9^/L, n (%)	10 (20%)
Leukocyte count (x 10^9^/L), median (range)	10.9 (2-102)
Leukocyte count >25 x 10^9^/L, n (%)	4 (8%)
Neutrophil count (x 10^9^/L), median (range)	7.0 (0.8-47.3)
Blasts (%), median (range)	0.5 (0-6.2)
Blasts >1%, n (%)	21 (42%)
Constitutional symptoms, n (%)	21 (42%)
C-reactive protein (ng/mL), median (range)	2102 (47-12909)
Cell-free DNA (μg/mL), median (range)	0.55 (0.29-1.88)
LDH (U/L), median (range)	573 (204-2000)
DIPSS risk score, n (%)	
low	10 (20%)
intermediate-1	15 (30%)
intermediate-2	16 (32%)
high	9 (18%)
MIPSS70 risk score, n (%)	
low	5 (10%)
intermediate	28 (56%)
high	17 (34%)
Treatment, n (%)	
None	32 (64%)
Hydroxyurea***	7 (14%)
Ruxolitinib	11 (22%)
Leukemic transformation, n (%)	5 (10%)
Death, n (%)	18 (36%)
Follow-up (months), median (range)	33 (1-104)

*High molecular risk mutations were identified in 19 (38%) patients, 3 of them with ≥2 variants. ASXL1 comprised frameshift or nonsense variants while the remaining variants included missense substitutions in IDH2 (p.R140Q), SRSF2 (p.P95H/L/R, p.D97Y) and U2AF1 (p.Q157P).

**Unfavorable karyotype was considered as any abnormal karyotype other than normal karyotype or sole abnormalities of 13q−, +9, 20q−, chromosome 1 translocation/duplication or sex chromosome abnormality including -Y.

***1 patient was treated with alpha interferon in addition to hydroxyurea.

ET means essential thrombocythemia; PV, polycythemia vera; DIPSS, dynamic international prognostic scoring system; MIPSS70, Mutation-Enhanced International Prognostic Score System70.

C-reactive protein and cell-free DNA levels in controls were 205.2 (71.4-3228) ng/mL and 0.32 (0.16-0.47) μg/mL, respectively.

Reference values for LDH, 125-220 U/L.

Clinical and laboratory parameters were collected by chart review. Percentage of monocytes and blasts in peripheral blood were assessed by manual differential count. Driver and HRM mutations were assessed as described (30).

### Plasma samples

Plasma samples were obtained from EDTA-anticoagulated blood, processed by two sequential centrifugation steps at 1000x*g* at 4°C during 15 minutes each and stored at −80°C until analysis.

### Monocyte isolation and stimulation

Mononuclear cells were isolated from EDTA-anticoagulated peripheral blood by Ficoll density gradient centrifugation (Cytiva, Sweden). Then, monocytes were purified by positive selection by MACS^®^ Technology using CD14 microbeads (Miltenyi Biotec, Germany). Cell purity was routinely over 95%. After isolation, 10^6^/mL monocytes were seeded in RPMI-1640 medium with L-Glutamine, with phenol red (Life Technologies, NY, USA), supplemented with 10% human heat-inactivated serum obtained from a healthy individual, and penicillin/streptomycin (Life Technologies). A control sample was assayed in parallel with a patient sample in all cases. For stimulation, increasing concentrations of Lipopolysaccharide (LPS) derived from Escherichia coli O111:B4 (Sigma-Aldrich, St. Louis, MO, USA) was used as a TLR4 agonist (1 ng/mL, 10 ng/mL, 100 ng/mL) and Pam3CSK4 (InvivoGen, San Diego, CA, United States) as a TLR1/2 ligand (1 ng/mL, 10 ng/mL, 100 ng/mL). Untreated cells served as control. Cells were incubated for 4 h at 37°C, 5%CO2, in a humidified atmosphere and RNA was extracted using Trizol reagent (Life technologies, Carlsbad, CA, USA) and reverse transcribed using SuperScript II Reverse Transcriptase (Thermo Fisher Scientific, Carlsbad, CA, USA). cDNA was analyzed by qRT-PCR using SYBR^®^ Green (LifeTechnologies, NY, USA) in a CFX96 Connect Real-Time PCR Detection System (BioRad, USA).

In another set of experiments, 10^6^/mL monocytes from each patient/control sample were seeded in a 24-well plate and incubated alone or with 100 ng/mL of LPS. Supernatants were harvested after 20 h, centrifuged at 1000 x *g* and stored at −80°C until analysis.

### Quantification of DAMPs in plasma samples and monocyte culture supernatants

S100A8/A9 was measured by a Quantikine, Human S100A8/S100A9 heterodimer ELISA (R&D Systems, Minneapolis, MN, USA) and HMGB1 by Human HMGB-1 ELISA (Novus Biological, CO, USA) in plasma or monocyte culture supernatants according to the recommendations of the manufacturer.

### Quantification of proinflammatory cytokines IL-1β and IL-6

Monocyte expression of proinflammatory cytokines IL-1β and IL-6 was assessed in purified monocytes by real-time reverse transcription polymerase chain reaction at baseline or after stimulation with TLR1/2 and TLR4 agonists, as mentioned above. Primer sequences were: IL-1β foward: 5’-AAACCTCTTCGAGGCACAAG -3’; IL-1β Reverse: 5’-GTTTAGGGCCATCAGCTTCA -3’; IL-6 forward: 5’-AGTAGTGAGGAACAAGCCAGAG-3’; IL-6 reverse: 5’- CAGGGGTGGTTATTGCATCT-3’. Samples were run in triplicate and assessed relative to glyceraldehyde-3-phosphate dehydrogenase (GAPDH).

IL-1β and IL-6 were also measured in plasma samples by Quantikine HS ELISA for Human IL-6 or Human IL-1β/IL-1F2 (R&D Systems, Minneapolis, MN, USA), according to the recommendations of the manufacturer.

### Incubation of healthy monocytes with patient or control plasma

Purified monocytes (10^6^/mL) isolated from peripheral blood from healthy controls (0 positive blood type) were incubated in RPMI-1640 supplemented with 10 UI/mL heparin in the presence of 10% plasma samples from patients (n=15) or controls (n=10) during 4 h at 37°C, 5%CO2, in a humidified atmosphere. After centrifugation, monocytes were lysed in Trizol followed by RNA extraction and 50 ng RNA were reverse transcribed using SuperScript VILO (Life Technologies). Gene expression of IL-1β and IL-6 was assessed by qPCR, as described previously.

### Monocyte surface TLR2 and TLR4 expression

TLR2 and TLR4 expression was measured in EDTA-anticoagulated whole blood diluted with phosphate buffer saline (PBS) and adjusted to 35 x 10^4^ leukocytes in the presence of FITC-conjugated anti-CD14 (BioLegend, San Diego, CA, USA), to identify the monocyte population, and PE-conjugated anti-TLR2 or anti-TLR4 (BD Biosciences, San Jose, CA, United States) for 30 min at room temperature. Cells were fixed with 1% paraformaldehyde (PFA) and analyzed by flow cytometry. A control sample was run in parallel with a patient sample in all cases. Mean fluorescence intensity of TLR staining relative to the corresponding isotypic control was expressed as relative fluorescence intensity (RFI).

### Monocyte functional responses triggered by TLR4 and TLR2 stimulation

To assess adhesion molecule CD11b expression, heparin-anticoagulated whole blood was incubated with FITC-conjugated anti-CD14 and PE-conjugated anti-CD11b (BioLegend, San Diego, CA, USA) at baseline or after stimulation with growing concentrations of LPS (0.1 ng/mL, 1 ng/mL, 10 ng/mL) and Pam3CSK4 (1 ng/mL, 10 ng/mL, 100 ng/mL) for 15 min at 37°C. Next, cells were incubated for 10 min in lysis buffer (BD Biosciences, San Jose, CA, USA), washed, fixed with 1% PFA and analyzed in a flow cytometer.

Tissue factor expression was assessed in heparin-anticoagulated whole blood that was incubated with FITC-conjugated anti-CD14 and PE-conjugated anti-CD142 (BD Biosciences, San Jose, CA, USA) at baseline or after stimulation with increasing concentrations of LPS (0.1 ng/mL, 1 ng/mL, 10 ng/mL) and Pam3CSK4 (1 ng/mL, 10 ng/mL, 100 ng/mL) for 4 h at 37°C. Next, cells were incubated for 10 min in lysis buffer, washed, fixed with 1% PFA and analyzed in a flow cytometer.

In both experiments, the monocyte population was selected according to CD14 expression and side scatter. Mean fluorescence intensity of CD11b or tissue factor PE-staining was assessed in the CD14-positive population. A control sample was run in parallel with a patient sample in all cases.

### Statistical analysis

Data were tested for Gaussian distribution. For comparison between two groups, Mann-Whitney test, paired t test and Wilcoxon matched-pairs signed rank test were used. For comparison among multiple groups Kruskal-Wallis with *post hoc* Dunn´s Multiple Comparison test was used. Categorical values were examined by Fisher´s exact test and correlations, with the Spearman correlation test. Overall survival was calculated from the date of sample collection to the date of death (uncensored), last contact or stem cell transplantation (censored). Kaplan-Meier curves were generated and compared using the log-rank test. Multivariate analysis was performed using a Cox proportional hazard regression model. All statistical analyses were two-sided and P values < 0.05 were considered significant. The GraphPad Prism 9.3.1 (La Jolla, CA, USA) software and the STATA Software, 16.0 (College Station, TX, USA) were applied.

## Results

### Patients with myelofibrosis display elevated levels of damage-associated molecular patterns HMGB1 and S100A8/A9 in circulation

**Figure 1:**
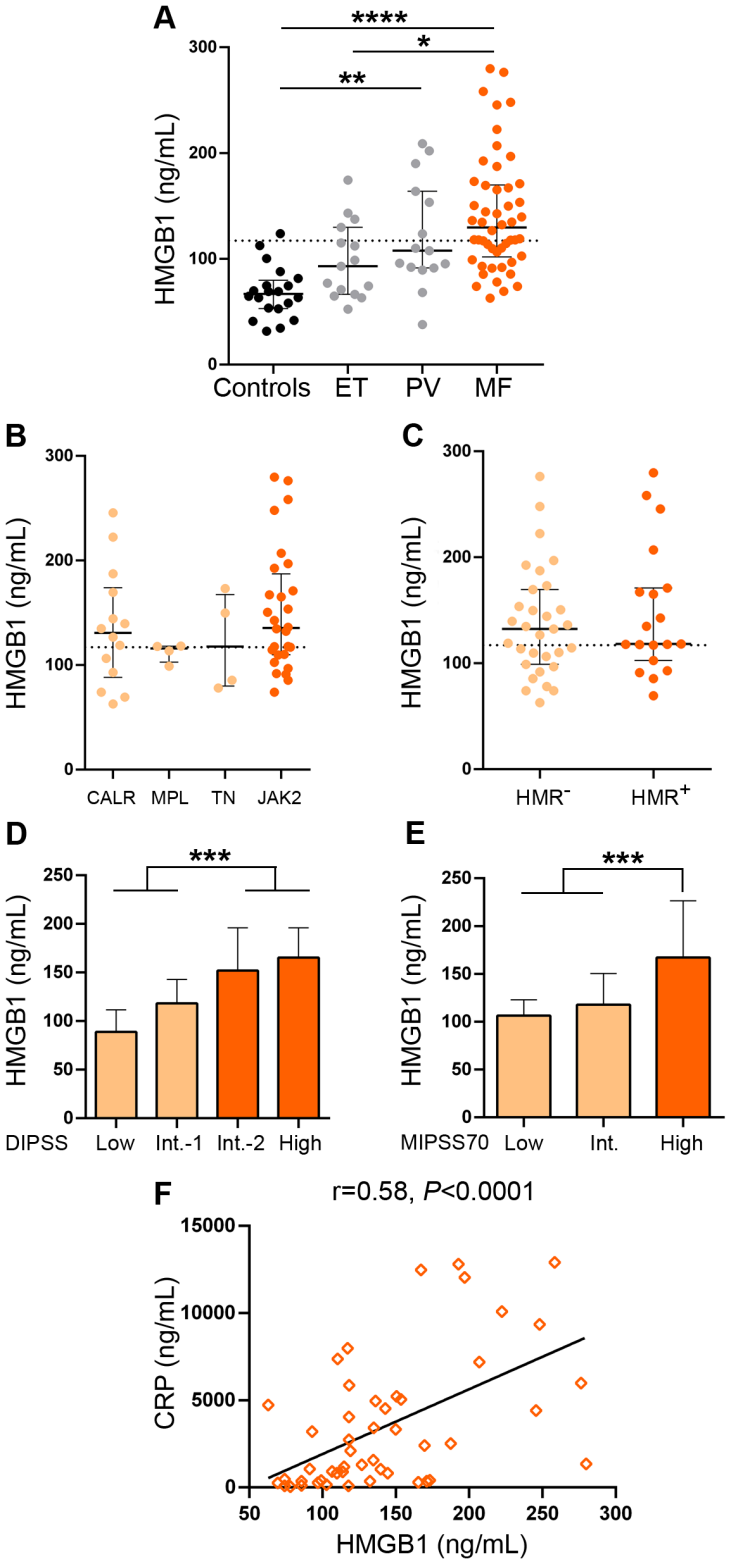
Levels of HMGB1 in patients with myelofibrosis. HMGB1 was measured in plasma by ELISA in **(A)** myelofibrosis (MF) (n=50), essential thrombocythemia (ET) (n=15) and polycythemia vera (PV) (n=15) patients and controls (n=20), *P<0.05, **P<0.01, ****P<0.0001, Kruskal-Wallis test. **(B)** MF patients grouped according to driver mutations, *CALR* (n=14), including CALR type 1 (n=13) and *CALR* type 2 (n=1), *MPL* (n=4), triple-negative (TN) (n=4) and *JAK2*V617F-positive (n=28) patients, *P*=NS, Kruskal-Wallis test. **(C)** MF patients positive (n=19) or negative (n=31) for high-molecular risk (HMR) mutations, *P=*NS, Mann-Whitney test. **(D)** MF patients stratified in low (n=10), intermediate (Int.)-1 (n=15), intermediate-2 (n=16) and high (n=9) risk groups according to the DIPSS score, ***P<0.001, Mann-Whitney test and **(E)** low (n=5), intermediate (n=28), and high (n=17) risk groups according to the MIPSS70 score, ***P<0.001, Mann-Whitney test. **(F)** Correlation between HMGB1 and C-reactive protein (CRP), *P*<0.0001, Spearman correlation. Dashed lines in **(A–C)** indicate reference values. Median with interquartile range values are shown.

Levels of HMGB1 in plasma were elevated in patients with MF compared to controls (**Figure 1A**). In particular, 62% of MF patients had levels above reference values, established as the mean +2SD of the control population. No significant differences were found between primary and secondary MF (**Supplementary Figure 1A**). Levels of this alarmin were also elevated in PV patients vs. controls, without showing significant differences when compared to the MF population, whereas HMGB1 concentrations in ET were mildly but not significantly elevated vs. controls and were lower than in MF (**Figure 1A**). Levels did not differ among MF patients grouped according to different driver mutation status (**Figure 1B**) or to the presence or absence of HMR mutations (**Figure 1C**). As previously shown for other inflammatory mediators, including several cytokines such as IL-8, IL-2R (5) and IL-18 (30), HMGB1 was higher in patients clustered in higher risk categories, as stratified by the DIPSS score, which is based on clinical variables (**Figure 1D**). To integrate clinical and genetic features, we then classified patients according to the MIPSS70 score. Although this prognostic system was originally developed for PMF patients age ≤ 70 years, it was also validated in patients with all ages (31) and has been recently shown to perform accurately in patients with secondary MF (32). Therefore, we applied the MIPSS70 score to our whole patient cohort, whose age ranged from 19 to 88 years old and included patients with secondary MF. As shown for the DIPSS prognostic model, HMGB1 levels increased from lower to higher MIPSS70 risk groups (**Figure 1E**), further emphasizing the relationship between levels of this alarmin and adverse risk factors. Although the number of patients with secondary MF patients was rather limited, a similar pattern was found when this subgroup of patients was classified according to the MYSEC-PM score (**Supplementary Figure S1C**). The DIPSS Plus and MIPSS70+v.2 prognostic systems were not used because cytogenetic analysis was not available for the whole patient cohort.

**Figure 2:**
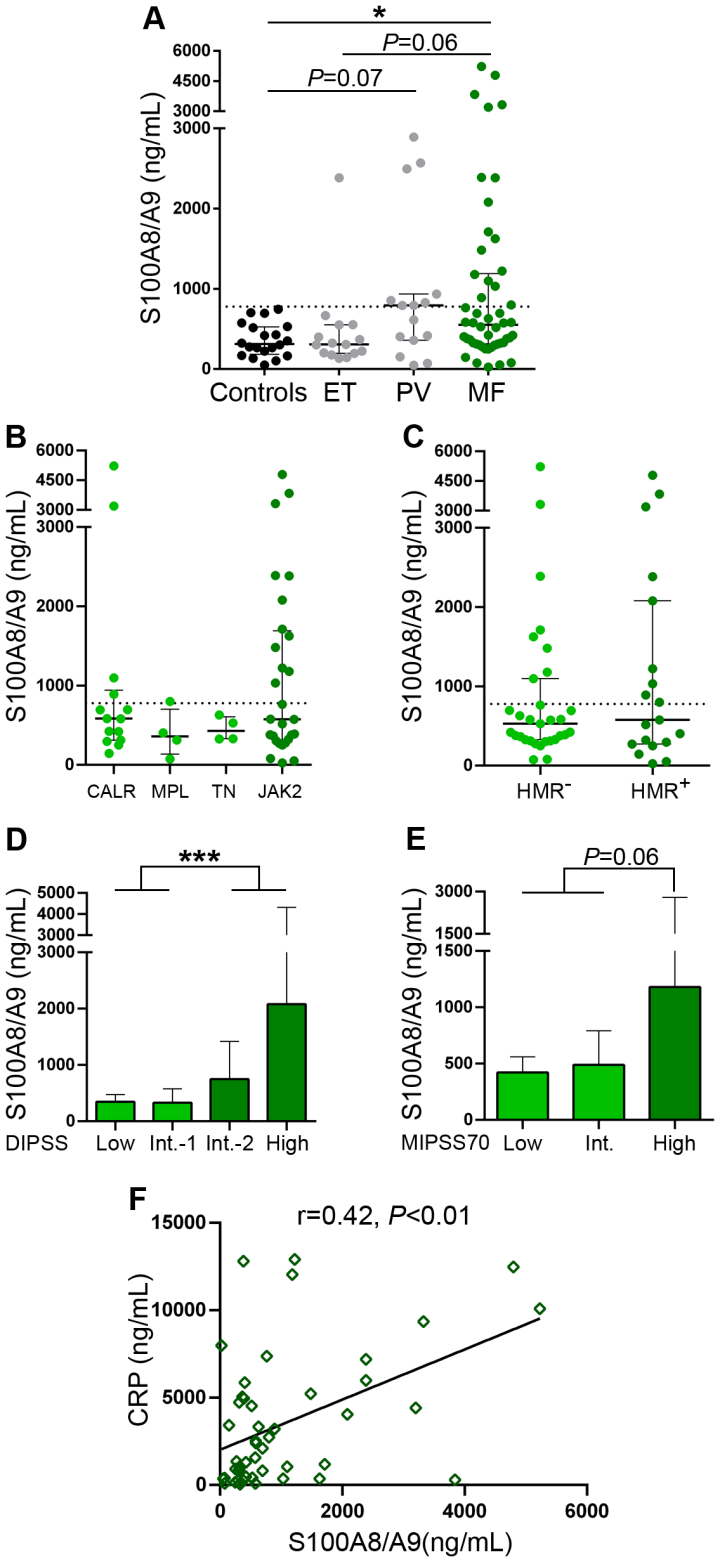
Levels of S100A8/A9 in patients with myelofibrosis. S100A8/A9 was measured in plasma by ELISA in **(A)** myelofibrosis (MF), essential thrombocythemia (ET) (n=15) and polycythemia vera (PV) (n=15) patients and controls (n=20), *P<0.05, Kruskal-Wallis test. **(B)** MF patients grouped according to driver mutations, *CALR* (n=14), including CALR type 1 (n=13) and *CALR* type 2 (n=1), *MPL* (n=4), triple-negative (TN) (n=4) and *JAK2*V617F-positive (n=28) patients, *P*=NS, Kruskal-Wallis test. **(C)** MF patients positive (n=19) or negative (n=31) for high-molecular risk (HMR) mutations, *P*=NS, Mann-Whitney test. **(D)** MF patients stratified in low (n=10), intermediate (Int.)-1 (n=15), intermediate-2 (n=16) and high (n=9) risk groups according to the DIPSS score, ***P<0.001, Mann-Whitney test and **(E)** low (n=5), intermediate (n=28), and high (n=17) risk groups according to the MIPSS70 score, *P=*0.06, Mann-Whitney test. **(F)** Correlation between S100A8/A9 and C-reactive protein (CRP), *P*<0.01, Spearman correlation. Dashed lines in **(A–C)** indicate reference values. Median with interquartile range values are shown.

The heterodimeric S100A8/A9 alarmin was also elevated in this MF cohort (**Figure 2A**), although to a lesser extent than HMGB1, as levels were above reference values in 34% of the patient population, without significant differences when primary and secondary MF were compared (**Supplementary Figure 1B**). S100A8/A9 values tended to be elevated also in PV, without significant differences when compared to MF, while levels in ET did not differ significantly vs. controls, tending to be lower than in MF patients (**Figure 2A**). As shown for HMGB1, no difference in S100A8/A9 levels was found among patients with different mutational profiles (**Figures 2B, C**), whereas levels of this alarmin were also found to be higher in patients classified into higher risk disease, as stratified according to the DIPSS score (**Figure 2D**) and a similar trend was shown for the MIPSS70 model (**Figure 2E**), whereas S100A8/A9 values were also higher in secondary MF patients belonging to higher risk MYSEC-PM categories (**Supplementary Figure S1D**).

Overall, 68% of patients had at least one of these DAMPs elevated. In fact, 28% of MF patients had concomitant elevation of both DAMPs, while 40% had increased levels of only one of them (34% had elevated HMGB1 alone and 6% had elevated S100A8/A9) and 32% had normal levels of both alarmins. Circulating levels of HMGB1 showed tight correlation with those of S100A8/A9 in the overall MF cohort (**Supplementary Figure 2**) and both DAMPs correlated with C-reactive protein, a marker of systemic inflammation previously shown to be elevated in this same patient cohort (30), (**Figures 1F**, **2F**), suggesting their participation in the generation of the inflammatory state.

**Figure 3:**
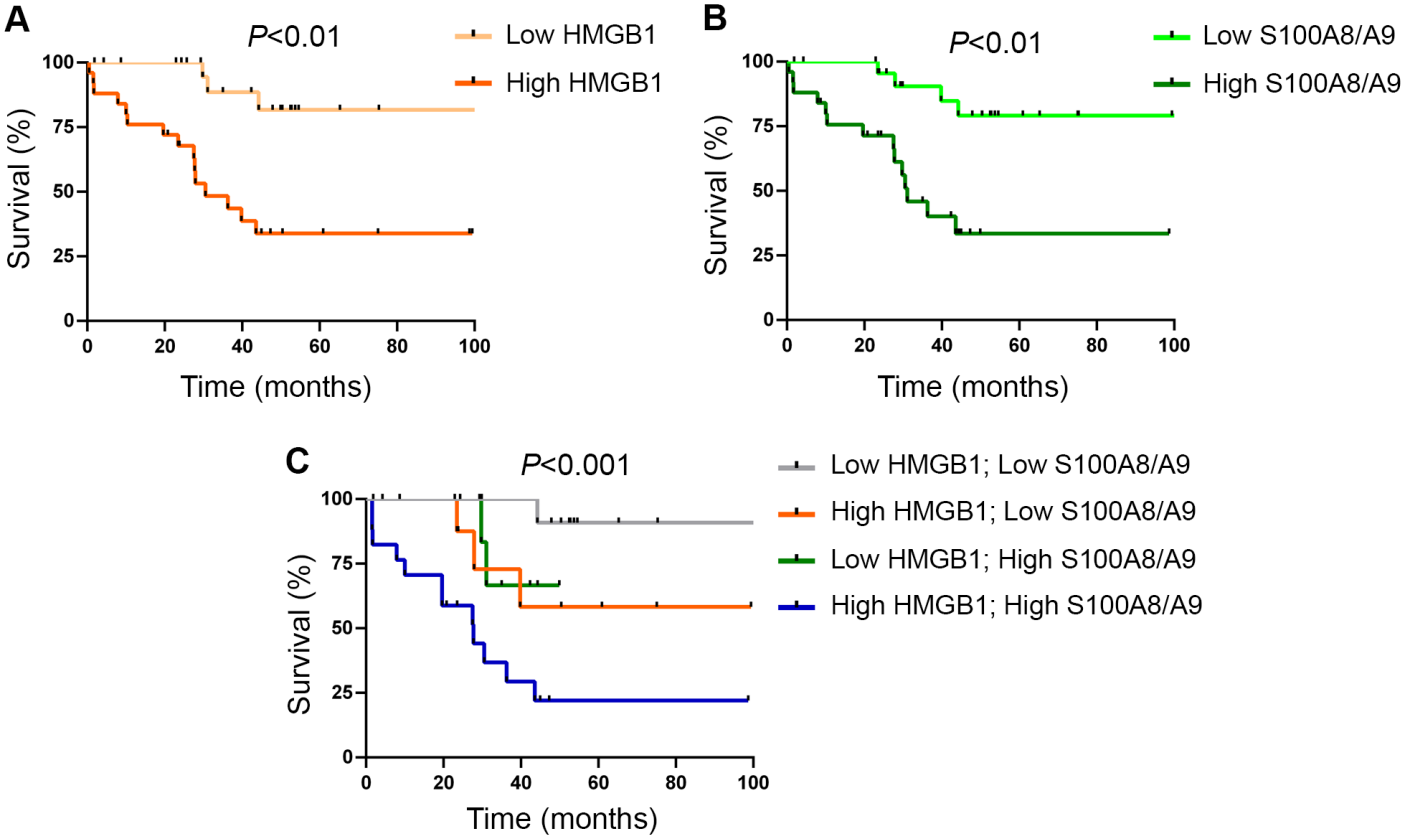
Survival analysis according to levels of circulating alarmins. **(A)** Overall survival in patients with myelofibrosis (MF) stratified according to high (upper two quartiles of the patient population) and low (lower two quartiles) HMGB1 levels, *P*<0.01, log rank test. **(B)** Overall survival in MF patients stratified according to high (upper two quartiles of the patient population) and low (lower two quartiles) S100A8/A9 levels, *P*<0.01, log rank test. **(C)** Combined survival analysis in MF patients grouped according to the presence of high or low levels of one or both alarmins, *P*<0.001, log rank test.

When MF patients were dicothomized according to DAMP levels, those with higher (upper two quartiles of the MF patient population) HMGB1 values had higher frequency of anemia (hemoglobin <10 g/dL), constitutional symptoms, a trend towards higher frequency of leukemic transformation (**Table 2**) and shorter overall survival, HR 6.7 (95%CI 1.9-23.2), *P*<0.01 (**Figure 3A**). With regards to S100A8/A9, patients with higher (upper two quartiles) plasma levels showed higher frequency of anemia, increased blasts (>1%) (**Table 2**) and inferior survival, HR 5.3 (95%CI 1.7-16.2), *P*<0.01 (**Figure 3B**). Interestingly, patients displaying higher levels of both alarmins (high HMGB1 + high S100A8/A9) showed inferior overall survival than those with isolated elevation of either alarmin (high HMGB1 + low S100A8/A9 or low HMGB1 + high S100A8/A9), or those with lower levels of both inflammatory mediators (low HMGB1 + low S100A8/A9), HR 21.2 (95%CI 2.7-163.6) for high HMGB1 + high S100A8/A9, *P*<0.001 (**Figure 3C**).

**Table 2:** Clinical features of MF patients according to HMGB1 and S100A8/A9 plasma levels.

	HMGB1					S100A8/A9				
	high (%)	low (%)	OR (95%CI)		*P*	high (%)	low (%)	OR (95%CI)		*P*
Hemoglobin <10 gr/dL	72	28	6.6	(1.9-20.1)	<0.01	72	28	6.6	(1.9-20.1)	<0.01
Transfusion need	28	12	2.9	(0.6-11.1)	NS	24	16	1.7	(0.5-5.8)	NS
Platelet count <100 x 10^9^/L	28	12	2.9	(0.6-11.1)	NS	20	20	1.0	(0.3-3.9)	NS
Leukocyte count >25 x 10^9^/L	16	0	–		NS	16	0	–		NS
Blasts >1%	44	40	1.2	(0.4-3.9)	NS	64	20	7.1	(1.9-24.4)	<0.01
Constitutional symptoms	68	32	4.5	(1.4-15.3)	<0.05	48	36	1.6	(0.5-4.7)	NS
Splenomegaly (>5cm LCM)	60	44	1.9	(0.6-5.4)	NS	56	44	1.6	(0.5-4.9)	NS
Leukemic transformation	20	0	-		0.05	16	4	4.6	(0.6-57.9)	NS

Patients with myelofibrosis (n=50) were dicothomized into those displaying high (upper two quartiles of the patient population) (n=25) vs. low (lower two quartiles) (n=25) levels of circulating HMGB1 and S100A8/A9. Clinical features were compared using Fisher´s exact test. NS, Not Significant.

To assess the independent significance of high HMGB1 and S100A8/A9 values on survival, we performed a Cox regression multivariate analysis including two widely used prognostic models. The effect of higher HMGB1 or S100A8/A9 levels on survival maintained its significance when adjusted for the MIPSS70 model, HR 3.9 (95%CI 1.1-13.8), *P*<0.05 for high HMGB1; HR 7.0 (95%CI 2.1-22.8), *P*<0.01 for high S100A8/A9, but its prognostic significance was no longer evident when analyzed in the context of the DIPSS classification, HR 3.0 (95%CI 0.8-10.9), *P*=NS for high HMGB1; HR 2.9 (95%CI 0.9-9.9), *P*=NS for high S100A8/A9. Of note, the effect of the concomitant elevation of both alarmins on survival proved to be independent of both the DIPSS and the MIPSS70 score, HR 3.7 (95%CI 1.2-11.6), *P<*0.05 and HR 6.3 (95%CI 2.3-17.6), *P<*0.001, respectively.

Median time elapsed from diagnosis to sample collection in this study was 43.9 (range 1-245.6) months and only 22% of patients were studied at the time of diagnosis. Prospective assessment of DAMP levels at diagnosis and sequential evaluation during follow-up, including at the time of leukemic transformation, would provide further information regarding their value as disease biomarkers. No significant differences in the levels of either DAMP were observed between patients without treatment and those receiving hydroxyurea or ruxolitinib in the overall patient cohort or when patients with lower and higher DIPSS risk scores were analyzed separately (**Supplementary Figure S3**). However, longitudinal measurement of alarmin levels in sequential samples obtained before and during treatment with hydroxyurea and ruxolitinb would be required to further determine the effect of these drugs on DAMP levels.

### Differential contribution of monocytes as a source of HMGB1 and S100A8/A9

**Figure 4:**
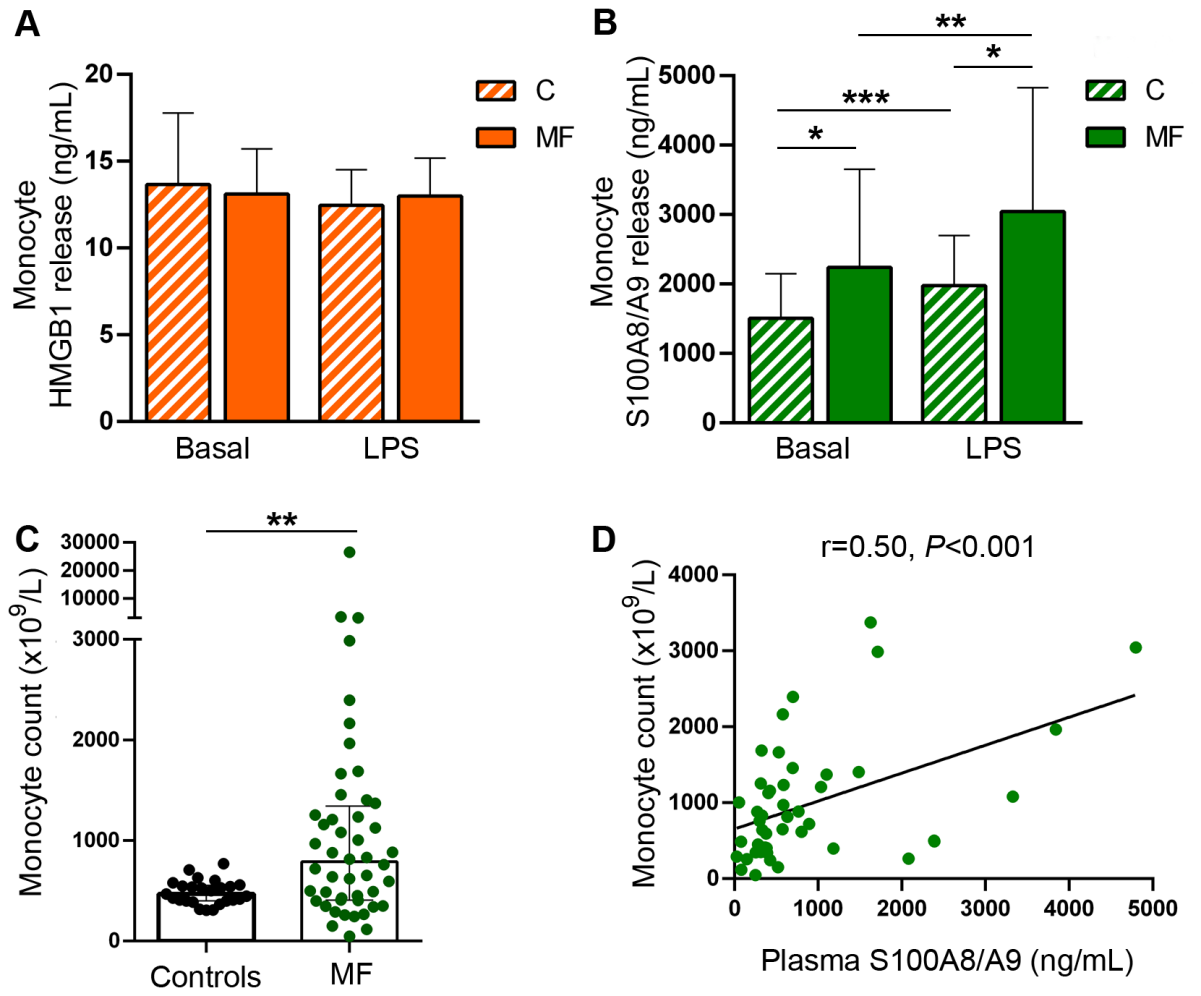
Monocyte release of damage-associated molecular patterns HMGB1 and S100A8/A9. Monocytes were purified from peripheral blood, cultured during 20 hours in basal conditions or stimulated with LPS 100 ng/mL and the release of HMGB1 and S100A8/A9 to the culture supernatant was measured by ELISA. **(A)** HMGB1 release in patients with myelofibrosis (MF) and controls **(C)** (n=10). **(B)** S100A8/A9 release in patients and controls (n=21), *P<0.05, **P<0.01, *** P<0.001 paired student *t* test. **(C)** Absolute monocyte count in patients and controls. **P<0.01, Mann-Whitney test **(D)** Correlation between monocyte counts and S100A8/A9 plasma levels in patients (n=48), *P*<0.001, Spearman correlation.

**Figure 5:**
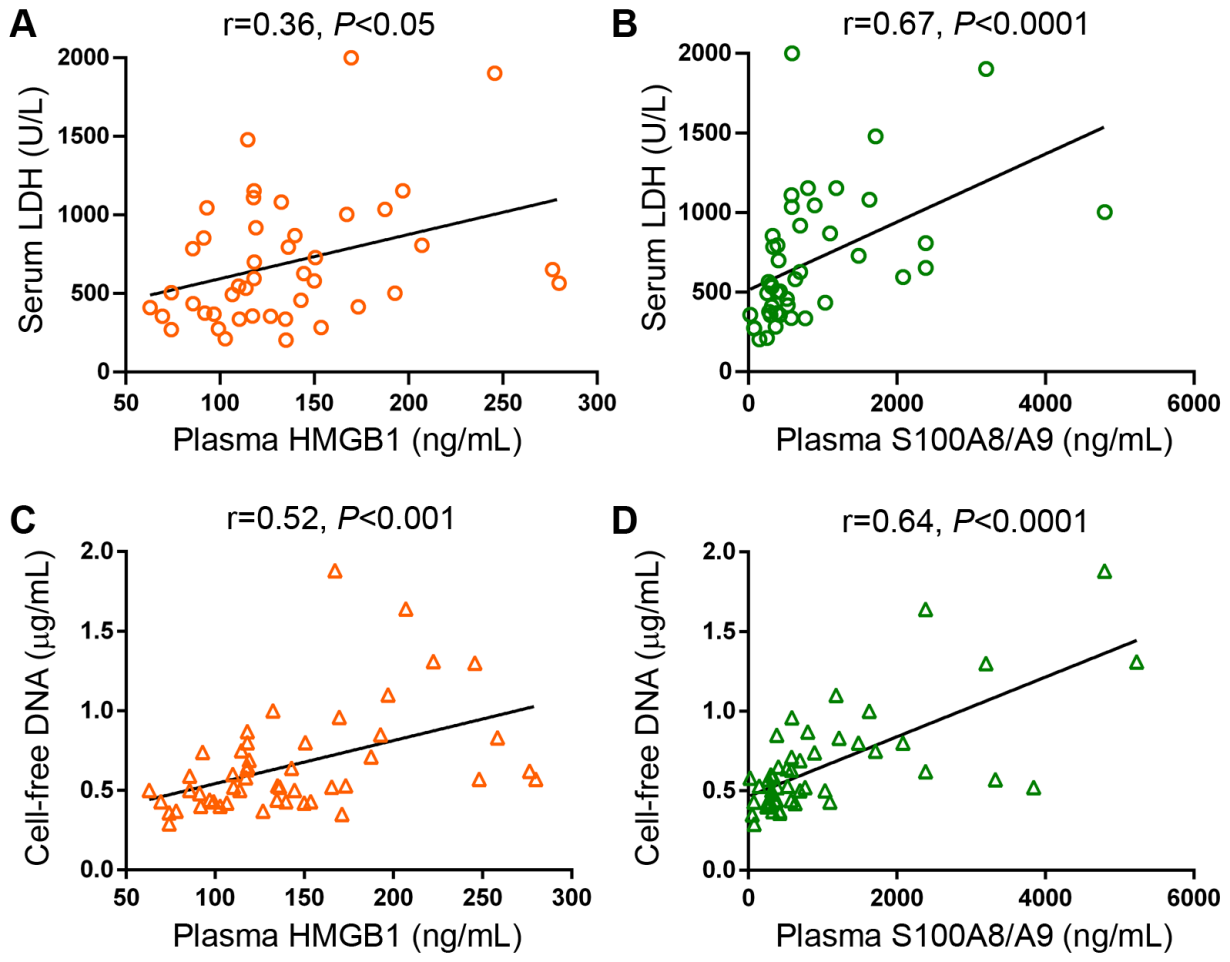
Correlation between levels of circulating HMGB1 and S100A8/A9 and cell death parameters in patients with myelofibrosis. Correlation between serum LDH and **(A)** HMGB1 and **(B)** S100A8/A9 plasma levels. Correlation between cell-free DNA and **(C)** HMGB1 and **(D)** S100A8/A9 plasma levels (n=50), *P*<0.05, *P*<0.001, *P*<0.0001, Spearman correlation.

Monocytes represent a major source of soluble inflammatory mediators in MF patients (10, 11). Considering that monocytes from healthy individuals have been shown to release HMGB1 and S100A8/A9 under steady state conditions and that LPS further stimulates the secretion of these alarmins (33–35), we assessed the contribution of these cells to the increase in circulating DAMPs in MF by measuring their release from purified monocytes to the culture supernatant after a 20-hour incubation period. Basal HMGB1 release did not differ between patients and controls, whereas, under our experimental conditions, LPS did not lead to consistent increases in HMGB1 secretion from either patient or control monocytes (**Figure 4A**). On the other hand, MF monocytes released larger amounts of S100A8/A9 both at baseline and following LPS stimulation (**Figure 4B**). Considering that monocyte counts were increased in this patient cohort (**Figure 4C**) and that tight correlation was found between monocyte counts and plasma S100A8/A9 levels (**Figure 4D**), active release of S100A8/A9 from monocytes could represent a relevant source of this alarmin in MF. Of interest, plasma S100A8/A9 also correlated with total leukocyte counts (r=0.76, P<0.0001) and absolute neutrophil counts (r=0.74, P<0.0001), indicating that active release from patient neutrophils could represent another potential mechanism contributing to the systemic increase in S100A8/A9, and highlighting that, overall, leukocytes are a relevant source of this alarmin in MF. In contrast, patient monocytes did not release larger amounts of HMGB1 as compared to control monocytes and no correlation was shown between plasma HMGB1 and leukocyte, monocyte nor neutrophil counts. Furthermore, although platelets have been shown to express and release both HMGB1 and S100A8/A9 (23, 36), they do not seem to substantially contribute to plasma levels of DAMPs in MF, as no direct correlation was shown with platelet counts (data not shown). The cellular origin of HMGB1 thus remains elusive and might be derived from several cell types, consistent with the fact that it is ubiquitously expressed (18).In addition to active secretion, DAMPs can be released by passive mechanisms as a result of diverse forms of cell death, including necrosis, pyroptosis and NETosis (16, 22, 23). To assess the contribution of cell death to DAMPs release, we assessed their relationship with cell death parameters, such as serum LDH and circulating cell-free (cf) DNA, which we previoulsy showed to be elevated in this same MF cohort (30). Both HMGB1 and S100A8/A9 correlated with LDH and cfDNA (**Figures 5A-D**), indicating that cell turnover resulting from exacerbated myeloproliferation may contribute to increased levels of these alarmins.

### Preserved expression of toll-like receptors 2 and 4 in monocytes

**Figure 6:**
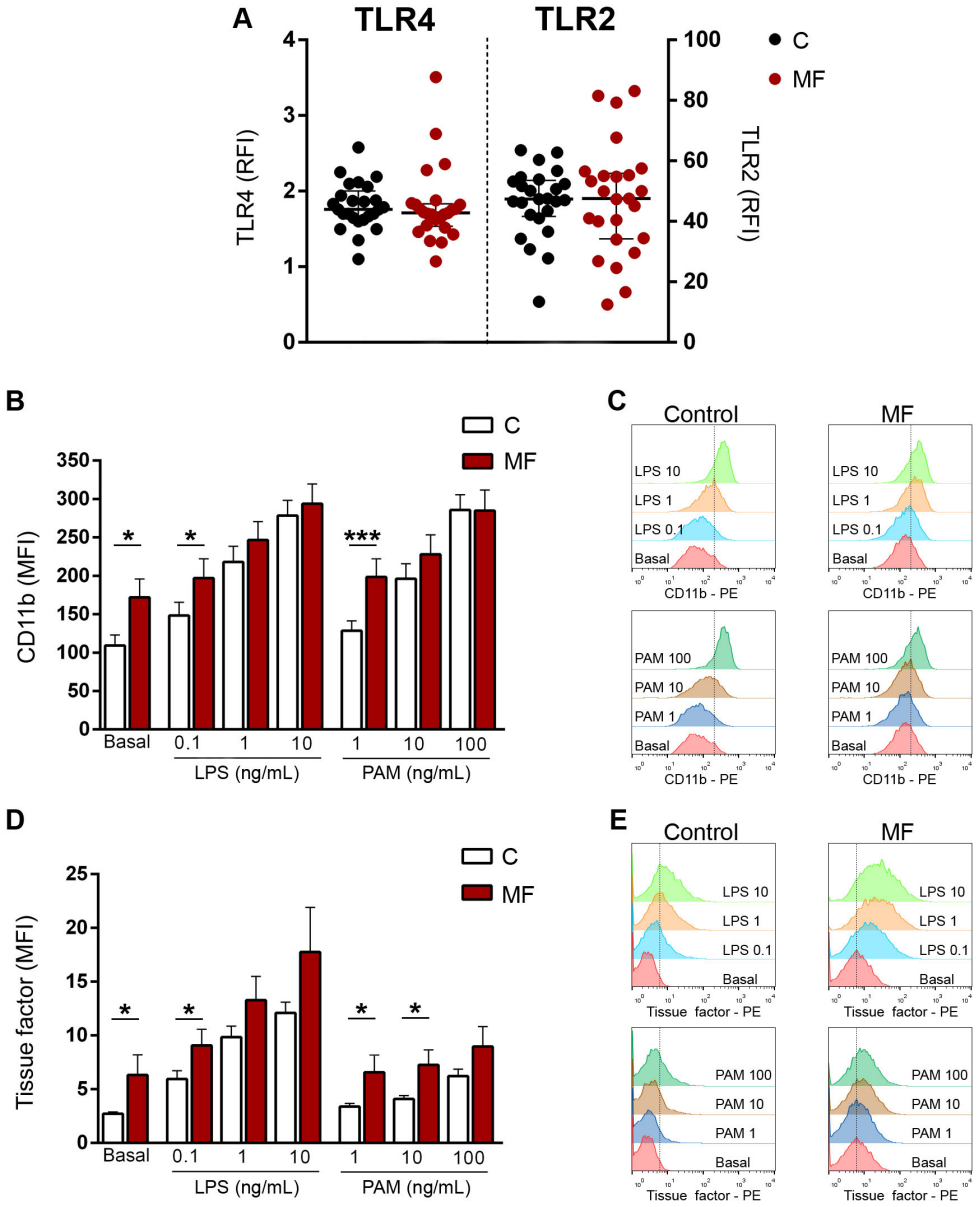
Expression of Toll-like receptors (TLR) 4 and TLR2 and TLR-triggered functional responses in monocytes. **(A)** Surface expression of TLR4 and TLR2 in monocytes from myelofibrosis (MF) patients (n=25) and controls (n=25) by flow cytometry. RFI, relative fluorescence intensity, *P*=NS, Wilcoxon test. **(B)** Monocytes from MF patients (n=25) and controls (n=25) were incubated with increasing concentrations of LPS or Pam3CSK4 (PAM) and surface expression of adhesion molecule CD11b was measured by flow cytometry. MFI, mean fluorescence intensity *P<0.05, ***P<0.001, Wilcoxon test for comparison between patients and controls. **(C)** A representative example of CD11b expression in MF and control monocytes is shown. **(D)** Monocytes from MF patients (n=25) and controls (n=25) were incubated with increasing concentrations of LPS or Pam3CSK4 and surface exposure of tissue factor was assessed by flow cytometry. *P<0.05, Wilcoxon test for comparison between patients and controls. **(E)** A representative example of tissue factor exposure in MF and control monocytes is shown.

Toll-like receptors (TLR) represent versatile sentinels of innate immunity that sense DAMPs in the setting of sterile inflammation (37). Both HMGB1 and S100A8/A9 activate TLR4, whereas HMGB1 also engages TLR2. Therefore, we first measured TLR4 and TLR2 expression in MF monocytes, which are key mediators of MF inflammation. Both TLR4 and TLR2 expression was preserved in monocytes from MF patients without differences when compared to healthy subjects (**Figure 6A**), indicating that these cells are able to adequately sense the increase in endogenous TLR ligands HMGB1 and S100A8/A9 found in circulation of MF patients.

### Monocyte expression of inflammatory markers triggered by TLR ligands

Next, to mimic the action of these DAMPs, we used two extensively characterized TLR ligands, such as lipopolysaccharide and the synthetic triacylated lipopeptide Pam3CSK4, which specifically engage TLR4 and TLR1/2, respectively, and assessed monocyte functional responses triggered by TLR4 and TLR1/2 stimulation, including adhesion molecule CD11b and tissue factor expression.

Monocyte activation leads to membrane expression of CD11b, which mediates monocyte adhesion to the endothelium and monocyte/platelet interaction, triggering the reciprocal activation of these cells, further inducing inflammation. Baseline CD11b levels were higher in patients than controls, reflecting spontaneous monocyte activation. Incubation with growing concentrations of LPS and Pam3CSK4 led to an increase in CD11b levels both in controls and patients. Patient monocytes reached higher CD11b levels upon stimulation with low and intermediate concentrations of both LPS and Pam3CSK4 as compared to controls, being significant at the lowest ligand concentrations. When higher concentrations of both agonists were used, the response of patients and controls reached similar levels. (**Figures 6B, C**).

Monocytes represent the main source of blood-borne tissue factor, which in addition to its classic role in initiating the clotting cascade, has well-established proinflammatory effects (38). As shown for CD11b, surface tissue factor on monocytes was elevated at baseline in patients vs. controls, reinforcing the fact that these cells are constitutively activated. Stimulation with all tested concentrations of LPS and, to a lesser extent, of Pam3CSK4 led to an increase in tissue factor membrane expression in control and patient monocytes. Patient monocytes achieved higher levels of tissue factor exposure following stimulation with both TLR agonists, reaching the level of significance for low LPS and low and intermediate Pam3CSK4 concentrations (**Figures 6D, E**).

### Monocyte expression levels of proinflammatory cytokines IL-1β and IL-6

**Figure 7:**
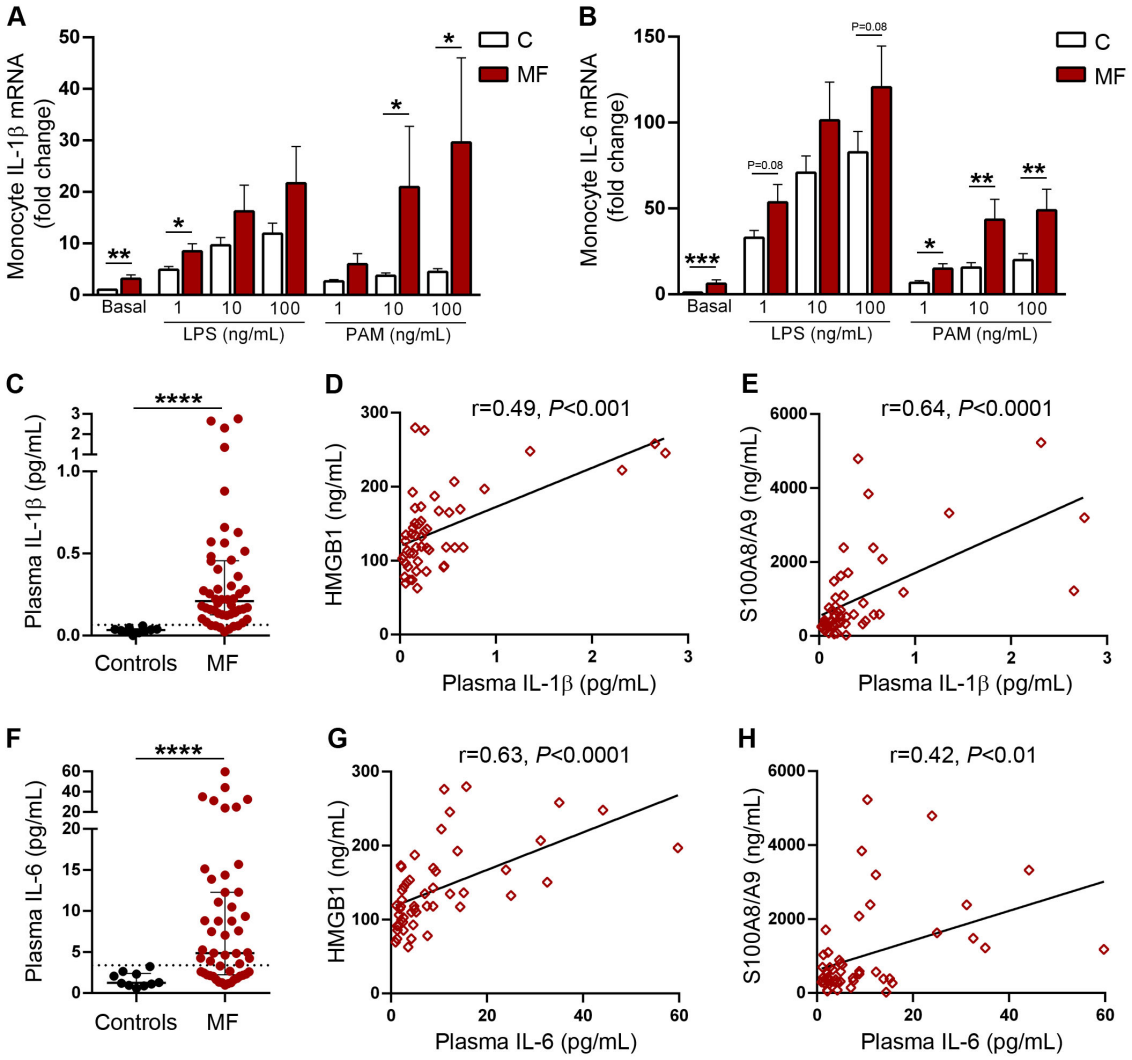
Monocyte gene expression and plasma levels of proinflammatory cytokines IL-1β and IL-6. Purified monocytes from patients with myelofibrosis (MF) (n=25) and controls (n=25) were incubated in the absence or presence of increasing concentrations of LPS or Pam3CSK4 (PAM) during 4 hs and gene expression of **(A)** IL-1β and **(B)** IL-6 was measured by qPCR. *P<0.05, **P<0.01 ***P<0.001 Wilcoxon test for comparison between patients and controls. Plasma levels of **(C)** IL-1β and **(F)** IL-6 in patients with MF (n=50) and controls (N=10) measured by ELISA, ****P<0.0001, Mann-Whitney test. Correlation between plasmatic IL-1β and circulating levels of **(D)** HMGB1 and **(E)** S100A8/A9. Correlation between plasmatic IL-6 and circulating levels of **(G)** HMGB1 and **(H)** S100A8/A9, *P*<0.01 *P*<0.001, *P*<0.0001, Spearman correlation.

Monocytes are a major reservoir of proinflammatory cytokines and can rapidly upregulate their synthesis upon stimulation with a wide array of inflammatory mediators which converge on NF-κB activation, including DAMPs (23, 25). To assess the contribution of TLR4 and TLR2 to cytokine production from monocytes, we studied baseline and TLR-stimulated gene expression of two inflammatory cytokines, IL-1β, which in addition to mediating vascular inflammation and bone marrow fibrosis, has been reported to promote clonal expansion of hematopoietic progenitors in MF (39, 40) and IL-6, a prototypic inflammatory cytokine. Baseline levels of IL-1β RNA were higher MF patients compared to controls, reflecting constitutive upregulation of cytokine expression. LPS stimulation induced an increase in IL-1β gene expression in both patients and controls. Levels tended to be higher in patients, reaching statistical significance at low LPS concentrations. Incubation with Pam3CSK4 also triggered an increase in IL-1β gene expression in patients and controls, albeit to a lesser degree than LPS. The response pattern in patients vs controls was similar to that of LPS, but in this case differences reached statistical significance at intermediate and high Pam3CSK4 concentrations (**Figure 7A**). As shown for IL-1β, basal IL-6 levels were higher in patients as compared to controls. Stimulation of TLR4, and to a lesser extent, TLR2 led to significant upregulation of cytokine transcription in both patients and controls. IL-6 mRNA levels tended to be higher at all LPS concentrations, although without reaching statistical significance, whereas differences induced by Pam3CSK4 were significant at all tested concentrations. (**Figure 7B**).

As an approach to determine whether elevated inflammatory mediators present in patient circulation could trigger upregulation of cytokine synthesis, we incubated monocytes isolated from healthy subjects with plasma samples obtained from patients harbouring elevated HMGB1 and/or S100A8/A9. Patient plasma samples induced higher IL-1β expression when compared to control plasma, reproducing findings elicited by TLR2 and TLR4 agonists, and a similar trend was shown for IL-6 (**Supplementary Figure S4**).

Finally, we measured systemic IL-1β and IL-6 concentrations. As previously reported (5, 39, 40), plasma levels of both cytokines were higher in MF patients as compared to healthy subjects (**Figures 7C, F**) and paralleled the degree of systemic inflammation, as reflected by their correlation with C-reactive protein levels (**Supplementary Figures S5A, D**). Levels of these inflammatory cytokines increased progressively in patients with more advanced disease stages, as stratified according to both the DIPSS and the MIPSS70 scores (**Supplementary Figures S5B, C, E, F**). Interestingly, circulating IL-1β and IL-6 showed a close relationship with both HMGB1 and S100A8/A9 plasma levels (**Figures 7D, E, G, H**), suggesting that common inflammatory signals, such as NF-κB, could induce concomitant upregulation of both DAMPs and proinflammatory cytokines. Additionally, the finding of elevated levels of HMGB1 and S100A8/A9 raises the possibility that these endogenous TLR ligands could contribute to exacerbated IL-1β and IL-6 production *in vivo* through stimulation of TLR-mediated signaling.

## Discussion

Dysregulated inflammatory response is a crucial aspect of MPN that contributes to disease pathogenesis and progression (1–4). This inflammatory state has been classically attributed to elevated levels of a dynamic spectrum of inflammatory cytokines, which have a well-established role in the disease (5, 10, 11). However, more recent evidence points to the participation of additional components of the innate immune system in MPN inflammation (14, 15). In this work, we show that two prototypical DAMPs, HMGB1 and S100A8/A9, which represent key players in innate immunity, are elevated in the circulation of MF patients. A gradient of HMGB1 and S100A8/A9 concentration was shown across patients with progressive disease stages, with lower levels in those with lower risk disease and higher levels in those with higher risk categories. These alarmins were also mildly elevated in plasma samples from ET patients, whereas patients with PV displayed intermediate values of HMGB1 and a consistent increase in S100A8/A9, overall indicating that levels of these DAMPs increase from early to more advanced MPN phenotypes.

This is, to our knowledge, the first study to identify increased levels of HMGB1 in patients with MF, revealing the participation of this DAMP in the inflammatory milieu that characterizes this neoplasm. On the other hand, the involvement of S100A8/A9 in MPN patients has been recently highlighted. Upregulation of the S100A8 and S100A9 subunits has been shown by gene expression and proteomic analysis of CD34^+^ cells and granulocytes from patients with all MPN subtypes (15, 41, 42). Subsequently, single-cell RNA sequencing of bone marrow cells displayed upregulation of the S100A8 subunit in mesenchymal stromal cells as a marker of fibrotic progression in MPN patients coupled with elevated S100A8 in circulation, whereas the S100A9 inhibitor tasquinimod ameliorated myeloproliferation and bone marrow fibrosis in a murine Jak2^V617F^ model, underscoring this alarmin axis as an attractive candidate for targeted therapy (43). In the aforementioned study, no correlation was found between plasma S100A8 and leukocyte counts, pointing to stromal cells as the main source of circulating S100A8 (43). In contrast, in our MF cohort plasma levels of S100A8/A9 showed tight correlation with total leukocyte counts and patient monocytes were shown to be an active source of S100A8/A9 release. Moreover, the close correlation between plasma S100A8/A9 and absolute neutrophil counts suggests neutrophil-derived S100A8/A9 could contribute to the systemic increase of this alarmin, although additional experiments should be undertaken to assess this possibility. In addition, CD34 cells may represent another potential source of S100A8/A9 in MF (42). Altogether, these results indicate that, in addition to the reported influence of stromal cells (43), leukocytes, and in particular monocytes, represent another relevant cellular source of S100A8/A9 in MF patients. In addition, passive release of both S100A8/A9 and HMGB1 induced by increased cell death secondary to clonal myeloproliferation could contribute to elevated systemic levels of these alarmins, as indicated by their association with cell death parameters.

Our data show that elevated DAMPs were associated with detrimental clinical features. In particular, high levels of HMGB1 and S100A8/A9 were strongly associated with anemia, which has a multifactorial pathogenesis and remains an unmet medical need in patients with MF. S100A8/A9 and HMGB1 have been shown to restrict erythropoiesis by suppressing EPO generation (44) or by interfering with EPO binding to its receptor (45), respectively, which could further contribute to anemia, providing a plausible explanation for the association of high DAMP signals and low hemoglobin values. In addition, HMGB1 and S100A8/A9 are potent triggers of NLRP3 inflammasome assembly (46, 47), providing a scaffold for caspase-1-mediated cleavage of Gasdermin D, which forms membrane pores leading to pyroptosis. Recently, S100A9-mediated induction of NLRP3 inflammasome-dependent pyroptotic cell death of hematopoietic progenitors has been shown to contribute to ineffective hematopoiesis and low blood counts in patients with myelodysplastic syndrome (MDS), whereas neutralization of S100A9 or pharmacologic NLRP3 inhibition were able to ameliorate pyroptosis and improve hemoglobin and platelet values in an MDS mouse model (48). Whether DAMP-triggered NLRP3 inflammasome activation and subsequent pyroptosis represents an additional factor contributing to anemia and cytopenias in MF, as demonstrated for MDS, remains to be determined. Interestingly, we recently showed that monocytes from MF patients display hyperactivation of the NLRP3 inflammasome in response to classic NLRP3 inducers, such as TLR4 ligand LPS and Nigericin (30). Future work would be required to assess whether this phenomenon also occurs in hematopoietic progenitor cells from MF patients, as described in MDS (48), which could subsequently lead to pyroptotic cell death of bone marrow precursors eventually contributing to ineffective hematopoiesis.

Several prognostic models based on clinical and genetic risk factors guide clinical decision making in MF (8). Our results revealed a negative impact of elevated HMGB1 and S100A8/A9 levels on survival. The predictive value of high levels of one of these two alarmins, either HMGB1 or S100A8/A9, on survival retained its significance when adjusted by the MIPSS70 but not the DIPSS score, whereas the combined effect of high levels of both HMGB1 and S100A8/A9, which was found in 34% of the study population, proved to be independent of both prognostic systems. Future studies including larger number of patients are warranted to further evaluate the impact of high levels of both alarmins as independent predictors of survival in MF and their potential value as prognostic indicators of disease outcome. If their predictive relevance is demonstrated in a larger independent cohort, DAMP levels could be considered as candidate biomarkers to be integrated into current prognostic models.

DAMP levels increased in parallel to the degree of systemic inflammation in this patient population, as reflected by their close correlation with C-reactive protein. HMGB1 and S100A8/A9 trigger inflammation, at least in part, by activating NF-κB through binding to TLRs expressed on diverse immune cells, including monocytes (18, 20, 21). To mimic the action of these endogenous TLR ligands and considering that HMGB1 activates TLR2 and TLR4, while S100A8/A9 binds TLR4, we used two well-characterized specific TLR4 and TLR1/2 agonists, such as LPS and Pam3CSK4, respectively (49) and assessed functional responses and cytokine upregulation in monocytes, which represent central players in innate immunity and the main source of cytokine production in MF (11, 50). As previously reported in several studies (11, 51, 52), monocytes from patients in this MF cohort were shown to be hyperactivated at baseline, as revealed by increased levels of cell activation markers, such as surface adhesion molecule CD11b and membrane exposure of tissue factor, and higher expression levels of two key proinflammatory cytokines, IL-1β and IL-6. Furthermore, patient monocytes displayed preserved or even exacerbated functional responses and cytokine upregulation following stimulation of TLR4 and TLR2. Dysregulated TLR signaling has been increasingly implicated in MF (11, 14, 49, 50). Our results extend findings from other groups showing exacerbated cytokine production from patient monocytes in response to TLR ligation. In this regard, Lai et al. demonstrated that TLR4 and TLR7/8 stimulation led to excessive TNF-α release from MPN monocytes, including ET, PV and MF, due to failure to dampen TLR signaling (50). Moreover, Fisher et al. showed that incubation of MF monocytes with TLR1/2 and TLR7/8 ligands induced the production of several cytokines, including TNF-α, IL-6, IL-1RA, IL-8 and MIP1β, some of them showing hypersensitivity to low doses of these ligands (11). Interestingly, we previously showed that platelets from ET patients display hyperreactivity to TLR4 and TLR2 ligation (49), denoting that this feature is not restrictive to MPN monocytes and might reflect a more generalized feature of MPN cells.

Altogether, elevated levels of endogenous TLR ligands HMGB1 and S100A8/A9 shown in the current study, coupled to the finding of preserved or hyperreactive TLR-triggered responses, suggest that DAMPs may promote monocyte activation and cytokine production in MF patients, thus fueling the inflammatory state (Graphical Abstract). The close correlation between circulating DAMPs and plasma levels of IL-1β and IL-6 raises the possibility that these alarmins could contribute to cytokine generation *in vivo*, although common inflammatory signals triggering concomitant upregulation of DAMPs and proinflammatory cytokines, such as NF-κB, could represent another plausible explanation. The pivotal role of IL-1β in MF progression has been recently highlighted by two independent studies, which showed elevated levels of IL-1β in patient samples, further demonstrating that interference with IL-1β signaling improves disease features and reverts bone marrow fibrosis in a murine MF model (39, 40). Our results confirm the finding of elevated IL-1β in patient circulation and reveal that IL-1β levels increase progressively along disease evolution, further strengthening the implication of IL-1β in disease progression. Similarly, IL-6 levels increased in parallel to the severity of the disease, reflecting that advanced disease is associated with higher degrees of inflammation, which in turn further fosters disease progression.

In this work, we used natural or synthetic bacterial components (LPS and Pam3CSK4) as TLR4 and TLR2 agonists in order to mimic the impact of DAMPs that signal through these pattern recognition receptors on monocyte activation. Interestingly, plasma samples from MF patients harbouring high levels of circulating alarmins triggered cytokine upregulation in healthy monocytes, suggesting that sterile inflammatory mediators are able to reproduce the effect shown in this study for LPS and Pam3CSK4. Further work will be required to assess whether primary patient monocytes show a similar behaviour when TLR4 and TLR2 are directly stimulated by DAMPs. In addition, genetic or pharmacologic approaches aimed at blocking or ablating these alarmins may help to definitively establish the role of the DAMP/TLR axis in monocyte activation and MF pathogenesis. In this regard, considering that tasquinimod has been shown to ameliorate bone marrow fibrosis and splenomegaly in JAK^V617F^ mice (43), it would be worth evaluating whether this S100A9 inhibitor is also able to block monocyte hyperactivation and lower cytokine levels in this model. Similar strategies using HMGB1 inhibitors, such as recombinant HMGB1 box A, would provide useful information regarding the participation of this alarmin in the inflammatory state.

In conclusion, this study highlights that, in cooperation with classic proinflammatory cytokines, DAMPs represent additional inflammatory mediators that may participate in the generation of MF inflammatory state. The presence of these endogenous TLR ligands may contribute to exacerbated TLR activation and TLR-triggered functional responses *in vivo*, further implicating dysregulated TLR signaling in MF pathogenesis. Targeting these inflammatory pathways may provide potential new therapeutic opportunities for MF patients.

## Data Availability

The raw data supporting the conclusions of this article will be made available by the authors, without undue reservation.
