# Supplementary material for: Elevated levels of damage-associated molecular patterns HMGB1 and S100A8/A9 coupled with toll-like receptor-triggered monocyte activation are associated with inflammation in patients with myelofibrosis
Source: Front Immunol. 2024 Sep 25;15:1365015. doi: 10.3389/fimmu.2024.1365015 (PMC11465240; doi:10.3389/fimmu.2024.1365015)
Supplement: Supplementary file 1 [file DataSheet1.pdf]

Supplementary Material

Supplementary Tables

Table S1. Features of patients with essential thrombocythemia and polycythemia vera.

	ET (n=15)	PV (n=15)
Age (years), median (range)	37 (22-80)	73 (31-92)
Female, n (%)	12 (80%)	8 (53%)
Driver mutation, n (%)		
JAK2 V617F	12 (80%)	15 (100%)
CALR type 1	2 (13%)	-
CALR type 2	-	-
MPL	-	-
Triple-negative	1 (7%)	-
Hemoglobin (gr/dL), median (range)	12,7 (10,1-15,4)	15,7 (12,7-16,5)
Platelet count (x 10^9/L), median (range)	656 (277-1311)	396 (246-1285)
Leukocyte count (x 10^9/L), median (range)	7,4 (3,7-7,2)	13,2 (5,8-37,3)
Thrombosis history, n (%)	5 (33%)	7 (47%)
Treatment, n (%)		
None	9 (60%)	9 (60%)
Hydroxyurea	6 (40%)	6 (40%)

ET means essential thrombocythemia; PV, polycythemia vera

Supplementary Figures

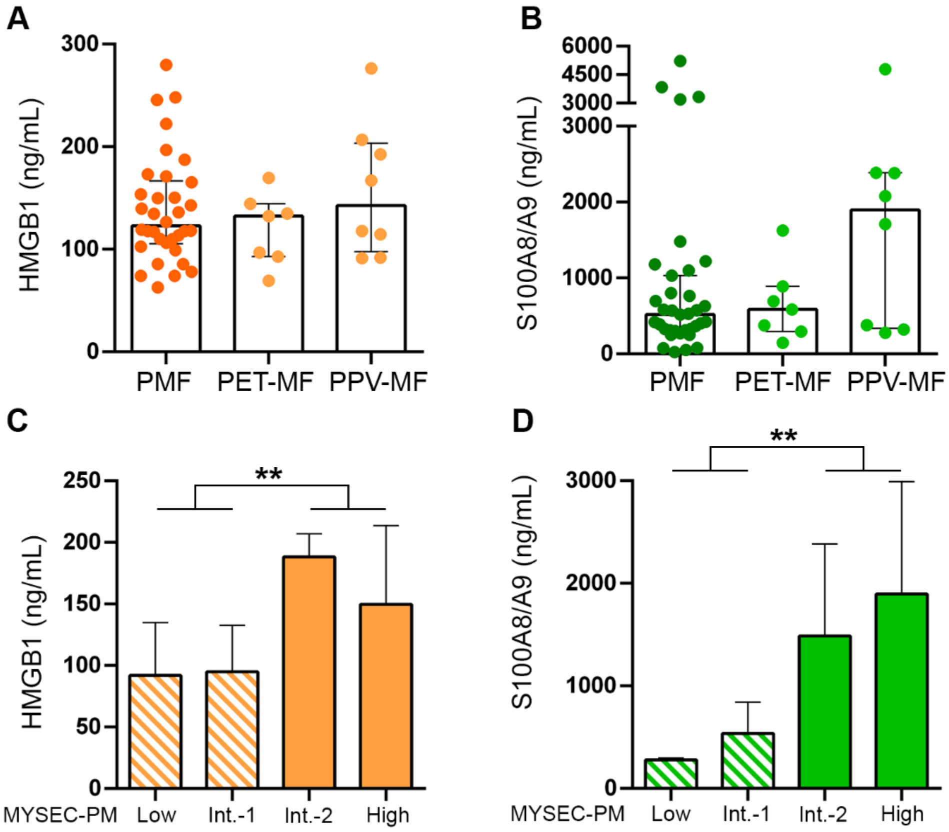

Figure S1. Levels of (A) HMGB1 and (B) S100A8/A9 in patients with primary (PMF) (n=35) and secondary myelofibrosis, including post-essential thrombocythemia (PET-MF) (n=7) and post-polycythemia vera (PPV-MF) (n=8) myelofibrosis. *P*=NS, Kruskal-Wallis test. Levels of (C) HMGB1 and (D) S100A8/A9 in secondary MF patients stratified in low (n=3), intermediate (Int.)-1 (n=4), intermediate-2 (n=2) and high (n=6) risk groups according to the MYSEC-PM score, ***P*<0.01, Mann-Whitney test.

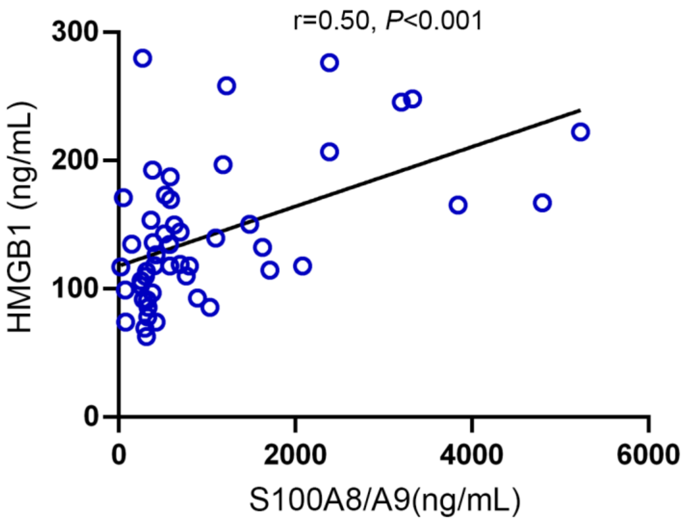

Figure S2. Correlation between levels of circulating HMGB1 and S100A8/A9 in patients with myelofibrosis (n=50), *P*<0.001, Spearman correlation.

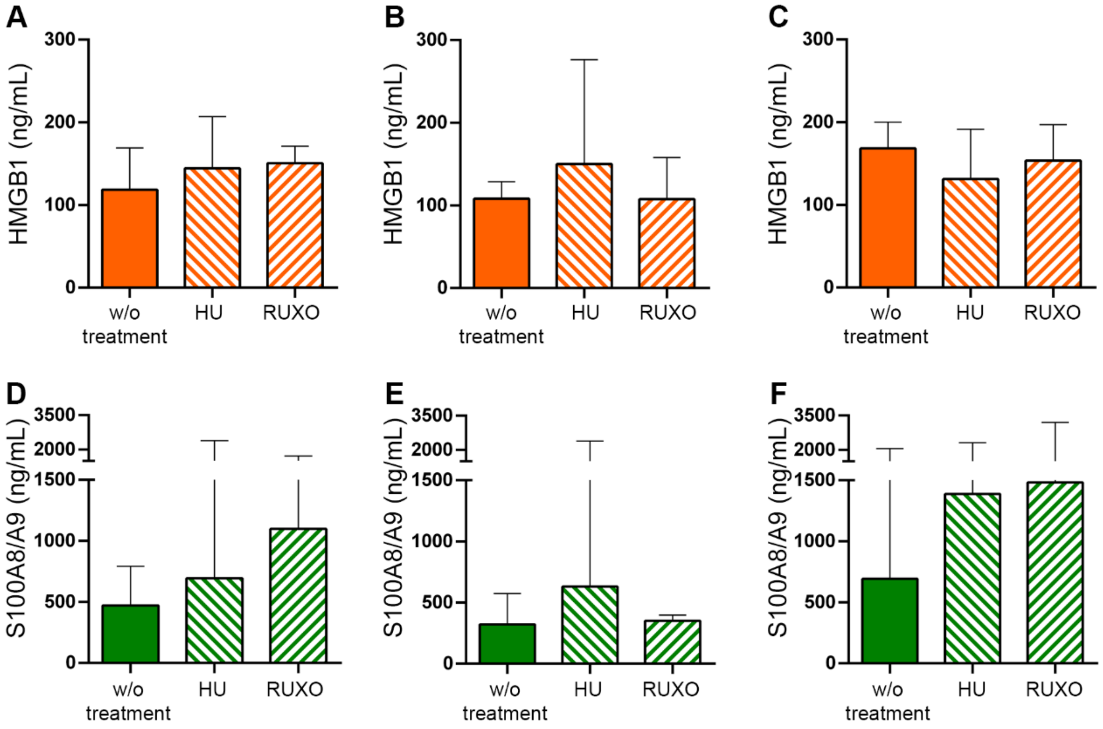

Figure S3. Circulating levels of HMGB1 and S100A8/A9 in patients with myelofibrosis (MF) without (w/o) treatment, treated with hydroxyurea (HU) or ruxolitinib (RUXO) in the overall cohort (n=50) (A, D), in patients with low and intermediate-1 DIPSS score (n=25) (B,E) and in patients with intermediate-2 and high DIPSS score (n=25) (C,F). Median and interquartile values are shown. *P*=NS, Kruskall-Wallis test.

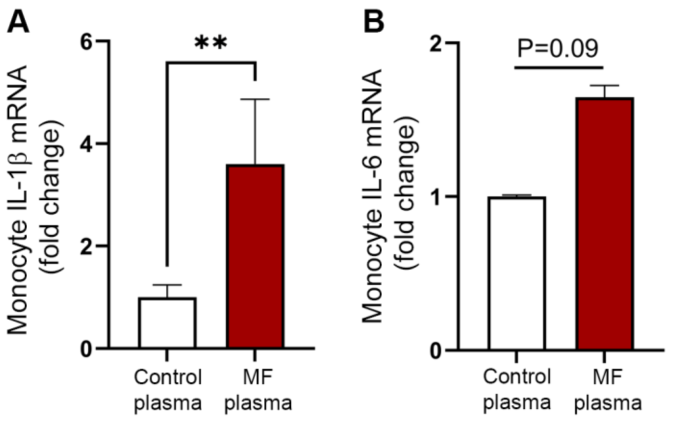

Figure S4. Monocytes from healthy controls were incubated with 10% patient or control plasma during 4 h and gene expression of (A) IL-1β and (B) IL-6 were measured by qPCR. ***P*<0.01, Mann-Whitney test.

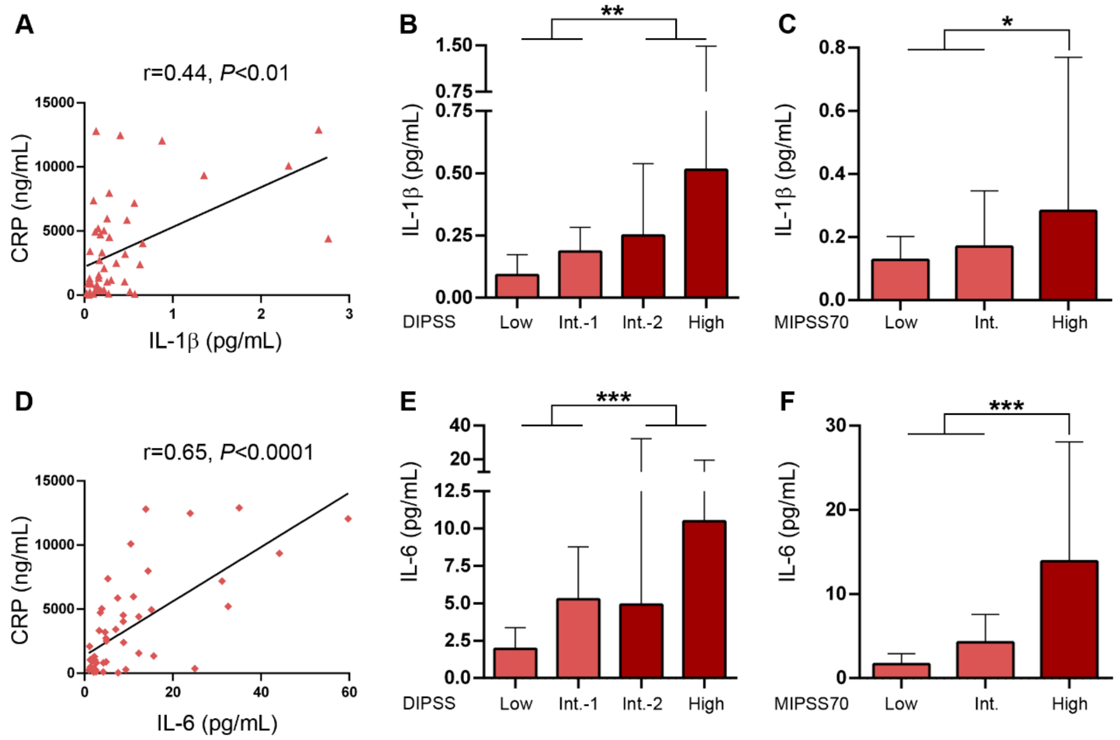

Figure S5. Correlation between plasma levels of IL-1β (A) and IL-6 (D) and C-reactive protein (CRP), *P*<0.01 and *P*<0.0001, Spearman correlation. Plasma levels of IL-1β (B,C) and IL-6 (E,F) in patients with myelofibrosis (MF) classified according to prognostic scores. Patients were stratified in low (n=10), intermediate-1 (n=15), intermediate-2 (n=16) and high (n=9) risk groups according to the DIPSS model and in low (n=5), intermediate (n=28) and high (n=17) risk groups according to the MIPSS70 score. Int (intermediate). Median and interquartile values are shown. **P*<0.05, ***P*<0.01, ****P*<0.001, Mann-Whitney test.
